# Differential Expression of NOTCH-1 and Its Molecular Targets in Response to Metronomic Followed by Conventional Therapy in a Patient with Advanced Triple-Negative Breast Cancer

**DOI:** 10.3390/biomedicines12020272

**Published:** 2024-01-25

**Authors:** Alice Ilari, Viola Cogliati, Noorhan Sherif, Emanuela Grassilli, Daniele Ramazzotti, Nicoletta Cordani, Giorgio Cazzaniga, Camillo Di Bella, Marialuisa Lavitrano, Marina Elena Cazzaniga, Maria Grazia Cerrito

**Affiliations:** 1 School of Medicine and Surgery, Milano-Bicocca University, 20900 Monza, Italy; a.ilari@campus.unimib.it (A.I.); n.sherif@campus.unimib.it (N.S.); emanuela.grassilli@unimib.it (E.G.); daniele.ramazzotti@unimib.it (D.R.); nicoletta.cordani@unimib.it (N.C.); marialuisa.lavitrano@unimib.it (M.L.); marina.cazzaniga@unimib.it (M.E.C.); 2 Phase 1 Research Centre, Fondazione IRCCS San Gerardo dei Tintori, Via Pergolesi 33, 20900 Monza, Italy; viola.cogliati@irccs-sangerardo.it; 3 Department of Pathology, Fondazione IRCCS San Gerardo dei Tintori, Via Pergolesi 33, 20900 Monza, Italy; giorgio9cazzaniga@gmail.com (G.C.); camillo.dibella@irccs-sangerardo.it (C.D.B.)

**Keywords:** metastatic triple-negative breast cancer (mTNBC), metronomic therapy (mCHT), cancer stem cells (CSCs), NOTCH-1, c-MYC, AKT

## Abstract

A group of 27 patients diagnosed with metastatic triple-negative breast cancer (mTNBC) was randomly distributed into two groups and underwent different lines of metronomic treatment (mCHT). The former group (N 14) received first-line mCHT and showed a higher overall survival rate than the second group (N 13), which underwent second-line mCHT. Analysis of one patient still alive from the first group, diagnosed with mTNBC in 2019, showed a complete metabolic response (CMR) after a composite approach implicating first-line mCHT followed by second-line epirubicin and third-line nab-paclitaxel, and was chosen for subsequent molecular characterization. We found altered expression in the cancer stemness-associated gene *NOTCH-1* and its corresponding protein. Additionally, we found changes in the expression of oncogenes, such as *MYC* and *AKT*, along with their respective proteins. Overall, our data suggest that a first-line treatment with mCHT followed by MTD might be effective by negatively regulating stemness traits usually associated with the emergence of drug resistance.

## 1. Introduction

Triple-negative breast cancer (TNBC) represents 10–20% of all breast cancers and is characterized by the absence of the expression of estrogen receptors (ER), progesterone receptors (PR), and the absence of amplification/over-expression of the human epidermal growth factor receptor 2 (HER2) [1]. These receptors are common targets for breast cancer therapies, and their absence in TNBC makes it more challenging to treat compared to other types of breast cancer. Chemotherapy and surgery have represented for years the only possible treatment approach for these patients [2], who very often relapse or have a spread of the tumor to other organs [3]. Currently, immune checkpoint inhibitors [4] and the new class of antibody–drug conjugates have provided valuable options for the treatment of TNBC [5,6]. Nevertheless, better therapeutic options are needed, both in terms of new drugs and methods of administering chemotherapy treatments. Many efforts are underway to identify new therapeutic targets and treatment strategies for TNBC.

Metronomic chemotherapy (mCHT), consisting of continuous administration of chemotherapy drugs at reduced doses, has been explored in selected patients as an alternative or a complement to conventional maximum tolerated dose regimens (MTD), where high doses of chemotherapy are administered for short periods of time followed by long periods of recovery [7]. Combining MTD and mCHT to potentially improve overall survival (OS) is a strategy that deserves further investigation. Our recent in vitro data demonstrated that mCHT administration of 5-FU+VNR is more effective than MTD schedule in controlling cell proliferation/survival and migration/invasion of both endothelial and TNBC cells, and has a strong antiangiogenic effect [8,9]. In this regard, more preclinical and clinical studies are required to establish a solid role for mCHT in cancer management [10].

Cancer stem cells (CSCs) are found in malignant tumors and share many resemblances with normal stem cells. They can self-renew and differentiate into numerous lineages, contributing to tumor growth and heterogeneity [11]. By supporting differentiation capacity, CSCs can drive the formation of heterogeneous tumors defined by the growth of phenotypically distinct subclones of transit-amplifying cells that accumulate and accelerate tumor growth [12]. In TNBC, pluripotency-related transcription factors, including OCT3/4, SOX2, NANOG, KLF4, and c-MYC, are overexpressed and correlate positively with poor prognosis, suggesting that the TNBC phenotype is remarkably similar to the CSC phenotype [13]. In addition, stemness circuits such as WNT/ß-catenin and NOTCH pathways are hyperactive in TNBC cells [14]. Furthermore, evidence suggests that therapy-resistant CSCs are increased in TNBC compared to other breast cancer subtypes, impacting therapy efficacy or patient outcomes, and significantly contributing to treatment failure, relapse, and increased mortality [15]. Indeed, deregulation of stemness pathways confers to TNBC CSCs (TNBCSCs), characteristics usually negatively correlated with response to chemotherapy, disease-free survival (DFS), metastasis-free survival, and OS [14]. Recent work [16] showed that NOTCH-1 has a key role in maintaining TNBCSC stemness; in fact, NOTCH-1 receptor expression and activation strongly correlate with the aggressive clinicopathological and biological phenotypes of breast cancer (e.g., invasiveness and chemoresistance), which are relevant characteristics of the TNBC subtype.

In the present study, we show that first-line mCHT allowed longer OS and DFS compared to mCHT used as a second line of treatment in a cohort of 27 female patients diagnosed with mTNBC and admitted at the IRCCS Fondazione San Gerardo dei Tintori (Monza, Italy). We conducted a study to examine the modulation of oncogenes such as *MYC* and *AKT*, and stemness markers such as *OCT3/4*, *SOX2*, and *NANOG*, coordinately activated by *NOTCH-1*, in one patient still alive after four years from the diagnosis of advanced TNBC and therapeutic approach consisting in first-line mCHT chemotherapy followed by MTD treatment.

## 2. Materials and Methods

### 2.1. Patients

**Figure 1:**
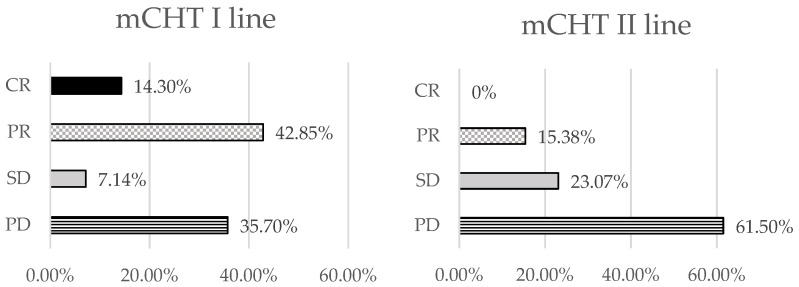
Responses of the patients after first-line (**left**) or second-line (**right**) mCHT. CR: complete response; PR: partial response; SD: stable disease; PD: progression disease.

A cohort of 27 patients diagnosed with metastatic TNBC (mTNBC) at the Medical Oncology Department and the Phase 1 Research Center of Fondazione IRCCS San Gerardo dei Tintori, Italy, was recruited from January 2011 to May 2022. All patients aged 18 or older, after the diagnosis of mTNBC, underwent either first-line mCHT treatment (N 14) followed by MTD chemotherapy or second-line mCHT treatment (N 13) after first-line treatment with MTD chemotherapy (Table 1 and Figure 1).

Among metronomic regimens, the preferred choice was the combination of oral vinorelbine (VRL) (Pierre Fabre Médicament, Paris, France) and capecitabine (CAPE), (Teva Pharmaceutical Industries, Milan, Italy) respectively, for 8 patients out of 14 (57.15%) in first-line mCHT subgroup and in 9 patients out of 13 (69.3%) in the second-line mCHT subgroup; oral VNR monotherapy was chosen respectively in 4 patients (28.6%) and 3 (23.1%), while only 2 patients (14.28%) in first-line mCHT subgroup and 1 (7.7%) in the second line one were treated with the association of VNR+CAPE+cyclophosphamide (CTX).

One patient still alive from the subgroup treated with first-line mCHT followed by MTD treatments was diagnosed with mTNBC, and showed both complete metabolic response (CMR) and clinically significant long and exceptional disease control for four years. Therefore, this patient was selected for a detailed analysis of clinical choices correlated with a deep molecular evaluation of the disease.

### 2.2. Patient Presentation

In 2011, a 62-year-old healthy woman received a diagnosis of invasive lobular carcinoma G2 after a superior-external quadrantectomy of the left breast, with a hormonal panel showing estrogen expression (ER) at 90%, progesterone (PgR) at 40%, Ki-67 at 3%, and the absence of HER2 overexpression. The pathological stage was pT1c pN0 (sentinel lymph node-negative). Subsequently, the patient underwent adjuvant hormonal therapy with letrozole for five years, combined with adjuvant radiotherapy.

**Figure 2:**
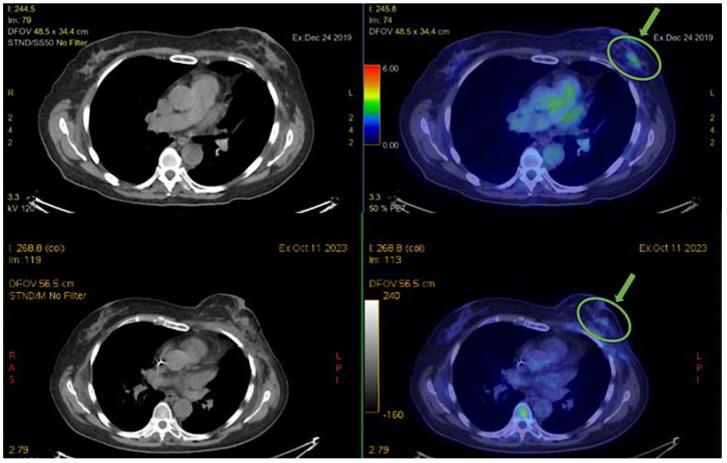
PET scan analysis before and after prolonged treatment. Top: 2019 PET scans; bottom: 2023 PET scans. The green arrow indicates the captured tracer and the ROI points to the region showing substantial disease control after 4 years.

After 8 years, in 2019, a recurrence of skin nodules at the site of previous breast surgery was diagnosed. A biopsy showed a significant change in the biology of the disease, with the loss of hormone receptor expression (ER/PgR 0/0%), while HER2 remained negative, and the level of Ki-67 was 25%. The systemic staging of the disease with an 18-18F-fluorodeoxyglucose (FDG)—positron emission tomography (PET) scan showed a weak uptake at the level of the known skin nodules and bilateral axillary lymph nodes (the top of the image of Figure 2).

In summary, we had a patient who was 68 years old, generally healthy, and without major underlying conditions. The patient was diagnosed with mTNBC, which had spread to the skin and lymph nodes. Unfortunately, the patient was not eligible for radical surgery with curative intent, making systemic treatments the next best option.

From January 2020, as first-line treatment, the patient begins an mCHT with the combination of oral VNR, CAPE, and CTX; thus, the patient is part of the first group mentioned above. The treatment was well tolerated, but later, a disease progression was evident both clinically and at the radiological assessment with PET. However, the disease still only affected the skin and bilateral axillary lymph nodes. At this point, a second-line treatment with standard CHT (epirubicin 30 mg/m^2^ for three weeks out of four) was started, with a complete response (CR) after 3 cycles, maintained after 6 total cycles. To optimizing the excellent response obtained, the patient underwent complementary radiation treatment to the left breast (for a dose of 40.05 Gy/15 fractions), completed in early 2021.

At the subsequent PET re-evaluation at the beginning of 2021, an excellent response was confirmed at the breast and left axillary levels. However, evidence of recurrence of active disease was found in the axillary and right subpectoral lymph nodes. Thus, a third line of standard systemic treatment was started with weekly nab-paclitaxel (Bristol Meyers Squibb, Rome, Italy). After 3 months of treatment, a partial response (PR) was obtained, while after 6 months, a complete metabolic response was evident, which was maintained for another 9 months when clinicians chose to interrupt this therapy and, for the second time, refer the patient to a personalized loco-regional approach, and radiotherapy treatment of right breast and ipsilateral axillary lymph nodes was performed (48 Gy/16 fractions). Substantial disease control was evident clinically and with instrumental re-evaluations of CT and PET scans (bottom of the image of Figure 2). Considering the unusual clinical course for a TNBC, a new biopsy under ultrasound guidance at the level of the left breast was performed. Anatomopathological analysis, however, confirmed the triple-negative tumor phenotype characterizing the patient, with a very low proliferation index (Ki-67 5%).

Given her good general condition, previous treatments, and, fortunately, the availability of new and innovative therapies, the patient started the antibody drug-conjugated sacituzumab govitecan (Gilead Sciences, Milano, Italy), with evidence of a new PR after 4 cycles, in October 2023 PET scans (bottom of the image of Figure 2). The treatment is still ongoing to date.

After administering mCHT treatment, we conducted a detailed molecular analysis to explore the characteristics of this disease and the effects of subsequent treatments.

### 2.3. Immunohistochemistry

Sections of 2 μm thickness were cut from tissue formalin-fixed, paraffin-embedded cancer tissue at the moment of the diagnosis of mTNBC and later at the recurrence and stored for the Department of Pathology, and stained for a panel of markers, including NOTCH-1, pAKT, c-MYC, p-21, SOX2, OCT3/4, NANOG, ER, PR, HER2, GATA3, and Ki-67 (primary antibodies used are listed in Table 2). The immunohistochemical evaluation involved the examination of immunostained sections by two breast pathologists. The evaluation included careful consideration of staining patterns, intensity, and distribution to derive insights into the molecular characteristics of the samples. The fields were scored as negative (<1% of the cells stained), + (1–19% staining), ++ (20–40% staining). Ki-67 and p21 were calculated as the percentage of positively stained tumor nuclei relative to overall tumor nuclei.

### 2.4. Data Source and Survival Analysis

This study utilized two publicly available data sets, representing cohorts of low-risk and high-risk patient survival, which are derived from the Cancer Genome Atlas (TCGA) and Molecular Taxonomy of Breast Cancer International Consortium (METABRIC).

A comprehensive analysis was conducted using regularized Cox regression with the LASSO penalty, generating coefficients for each considered gene. Positive coefficients (>0) indicated a positive association with risk, suggesting that the overexpression of these genes corresponds to a poor prognosis. Conversely, negative coefficients (<0) suggested a negative association with risk, implying that the downregulation of these genes is linked to a poor prognosis. This approach allowed for the evaluation of the impact of gene expression on the risk and prognosis of triple-negative breast cancer patients. Following the regularized Cox regression analysis, patient stratification was performed to create two distinct risk-based groups (high and low risk correlated with good and poor prognosis). This stratification was based on the risk estimates derived from the regression model. The objective was to obtain two clusters of patients exhibiting significantly different Kaplan–Meier survival estimates. This method allowed for a refined categorization of patients, enabling the identification of distinct survival patterns associated with varying levels of risk estimated through the regularized Cox regression methodology.

All bioinformatics analyses were performed using the R software version 4.3.2.

## 3. Results

### 3.1. mCHT Administered as First-Line Compared to Second-Line Therapy Increases the Therapeutic Response and Decreases the Progression of Disease in Patients with Advanced TNBC

To compare the effectiveness of mCHT administered as a first or second-line treatment in mTNBC patients, we first analyzed the objective response rate (ORR) of the two groups. ORR is defined as the percentage of patients who achieve a response while undergoing treatment, which may be a CR or a PR [17]. We observed that the ORR rate was 57.13% after first-line mCHT administration vs 15.38% when mCHT was given as a second-line treatment. A PR was observed in most of the patients (42.85%) during first-line mCHT, whereas the progression disease (PD) prevailed in second-line mCHT (61.50%) (Figure 1). Another important parameter that has been considered for evaluating the effectiveness of treatments is the time to progression (TTP), which is defined as the time from the start of the treatment until the first evidence of disease progression [18]. The median TTP was 7 months (range 1–37 months) for patients under mCHT therapy in first-line vs. a median TTP of 3 months (range 2–16 months) for those under mCHT therapy in second-line, thus indicating an obvious therapeutic benefit for the patients of the first group.

Although our study consists of a small pool of patients, we compared the OS of those treated with the first-line mCHT schedule and second-line MTD treatment (first group) with those treated in first-line with the MTD scheme and in the second-line with mCHT (second group). Median OS resulted differently in the first group compared to the second, with an average survival of the patients in the first group of 27 months vs. an average survival of the second group of 21 months. Furthermore, among patients undergoing mCHT in the first line, one patient was further characterized via instrumental examination and molecular analysis.

### 3.2. Instrumental and Molecular Characterization of a Patient Treated with First-Line mCHT

#### 3.2.1. PET Evaluations Show Prolonged Disease Control

The PET scan has acquired a fundamental role in the diagnosis of tumors, especially regarding TNBC. In fact, this technique has acquired a pivotal role as a biomarker for both cancer detection and disease progression [19]. Furthermore, this instrumental examination allows the identification of any occult distant metastases and any lymph node pathologies [20].

FDG-PET was performed to diagnose the patient’s breast cancer and then, during first-line mCHT and subsequent MTD chemotherapy, to follow the tumor’s response to therapy. A comparative analysis of the patient’s 2019 and 2023 PET images shows substantial stability/slight reduction of disease over a period of 4 years, from the diagnosis of metastatic disease to the present, highlighting the excellent control of the systemic disease obtained with the sequence of treatments carried out, as indicated by the green arrows and the ROI (region of interest) in Figure 2.

In addition to the parameters mentioned above, which are indicative of the effectiveness of the treatment (TTP, ORR, OS, and PET scan), there are also biological markers that empower the monitoring of the response to therapy. Among these markers, there is a growing interest in those related to stemness, which, when increased, are related to an unfavorable prognosis. We decided to analyze some of these markers to monitor the progress of the disease after treatments, as described below.

#### 3.2.2. Molecular Characterization of Advanced TNBC at the Diagnosis

**Figure 3:**
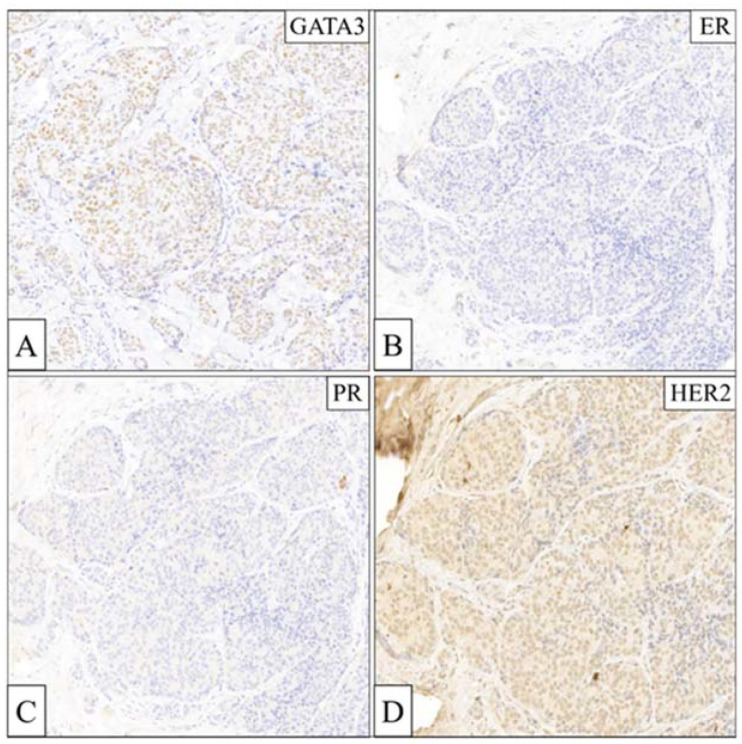
Molecular characterization of the mTNBC patient by immunohistochemistry staining (**A**). Nuclear GATA3 labeling, (**B**) labeling for ER-negative (<1%), (**C**) labeling for PR negative (<1%), (**D**) negative HER2 immunohistochemistry (20× magnification).

TNBC is classically defined for the lack of ER/PR/HER2 biomarkers, so its molecular characterization is challenging [21]. GATA3 expression can be used to identify of TNBC since it stains positive in around 85% of cases [22]. GATA3 in combination with ER, PR, and HER2 immunostainings resulted in the positivity of the first one and negativity of the other three, thus confirming the TNBC subtype of our case report (Figure 3A–D).

#### 3.2.3. Comparative Immunohistology of Stemness Markers at the Diagnosis of Advanced TNBC and at Recurrence

In addition to GATA3, the stemness marker NOTCH has also been recently suggested as a hallmark of TNBC [23]. NOTCH has a central role in stem cell biology and is a crucial player in cell-to-cell communications, regulating proliferation, self-renewal, and differentiation. NOTCH is a transmembrane receptor that, upon its ligand interaction, is cleaved, releasing an intracellular domain (NICD) that can be translocated into the nucleus or remain in the cytoplasm to crosstalk with proteins involved in other signaling pathways, such as c-MYC, AKT, and others.

**Figure 4:**
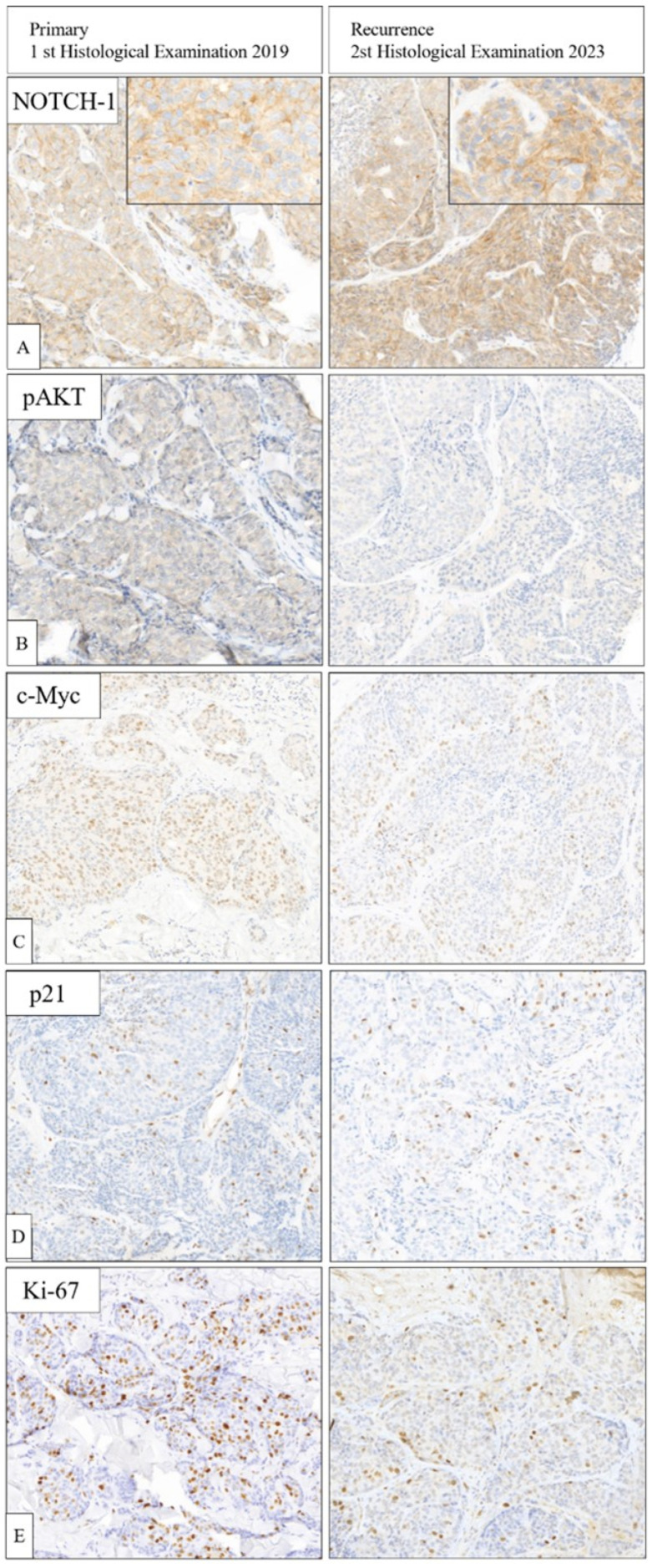
Comparative immunohistology between the histological evaluation at the time of diagnosis of mTNBC and at the recurrence. (**A**) NOTCH-1, (**B**) pAKT, (**C**) c-MYC, (**D**) p21, (**E**) Ki-67. (20× magnification, insert 40× magnification).

We compared the expression of NOTCH-1 and its downstream targets (AKT, c-MYC, OCT3/4, SOX2, NANOG) together with the cell cycle regulatory protein p21 and the proliferative marker Ki-67 in paired biopsies from the original tumor at the moment of the diagnosis (2019) with the more recent one (2023) after mCHT in first-line followed by MTD chemotherapy in second-line. As shown in Figure 4A, NOTCH-1 staining shifts from prevalent membranous (++) in the pretreatment sample to a combination of membranous (+) and cytoplasmic (+) in the posttreatment specimen.

The staining for the active phosphorylated form of AKT (pAKT), which was diffuse (+) in the first biopsy, completely disappeared in the last one (Figure 4B). A significant decrease in c-MYC staining was also observed (from ++ in 2019 to + in 2023) (Figure 4C). Accordingly, the cell cycle inhibitor p21, which is negatively regulated by c-MYC, is upregulated and its nuclear positivity increased from 6% in 2019 to 10% in 2023 (Figure 4E). In line with this, the proliferative marker Ki-67 expression strongly decreased from 25% before therapy to 5% after all cycles of treatment. Finally, OCT3/4, NANOG, and SOX2 were not expressed at the time of the diagnosis, nor in the recent biopsy.

#### 3.2.4. Association of *NOTCH-1*, *PIK3CA*, *AKT*, and *c-MYC* Expression with Prognosis

**Figure 5:**
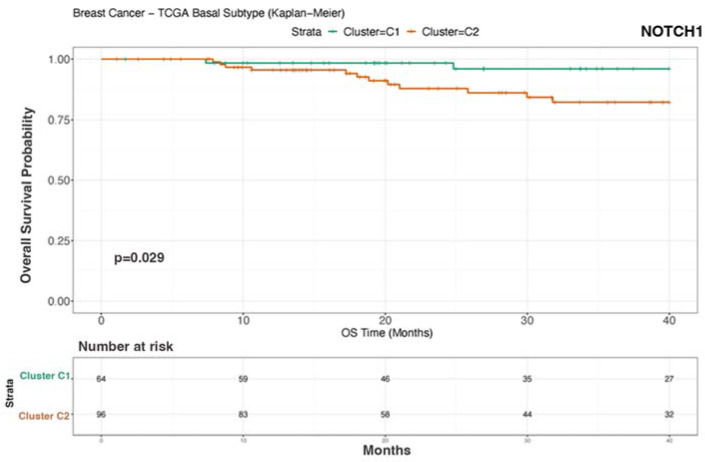
Kaplan–Meier survival analysis based on TCGA dataset. Cluster C1: good prognosis; Cluster C2: poor prognosis. TCGA analysis revealed that the patients with high *NOTCH-1* expression tended to have shorter survival (*p* = 0.029). TCGA, The Cancer Genome Atlas.

**Figure 6:**
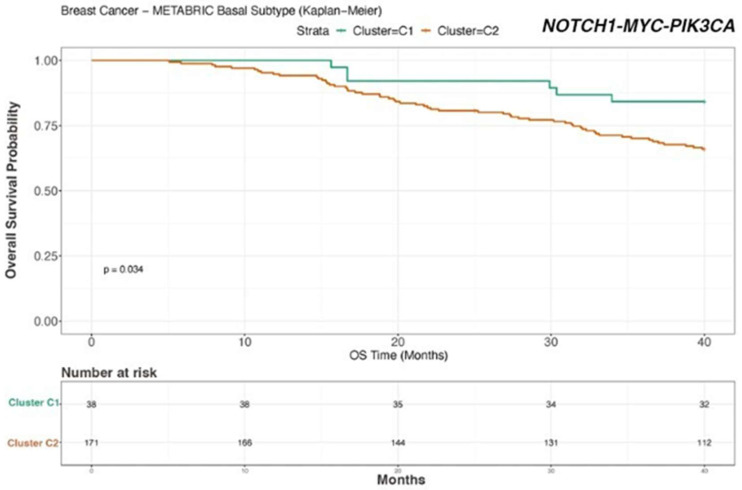
Kaplan–Meier survival analysis for the METABRIC dataset Cluster C1: good prognosis; Cluster C2: poor prognosis. METABRIC analysis revealed that the patients with high *NOTCH1*, *MYC*, and *PIK3CA* expression are correlated with shorter survival (*p* = 0.034).

**Figure 7:**
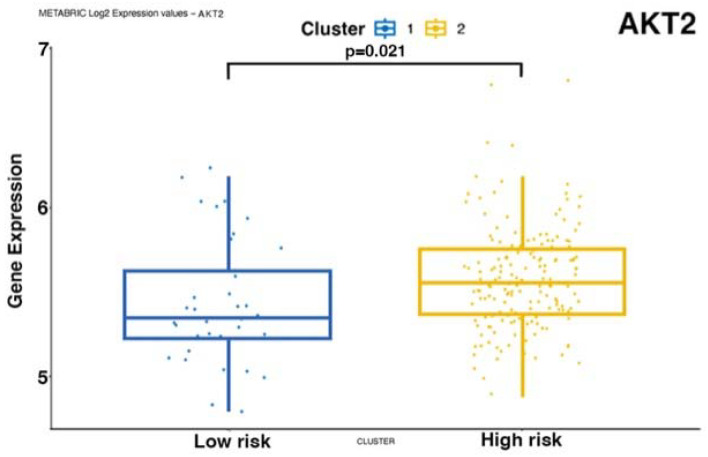
AKT2 gene expression in the two risk-associated clusters in the METABRIC dataset. Cluster 1: low risk; Cluster 2: high risk.

**Figure 8:**
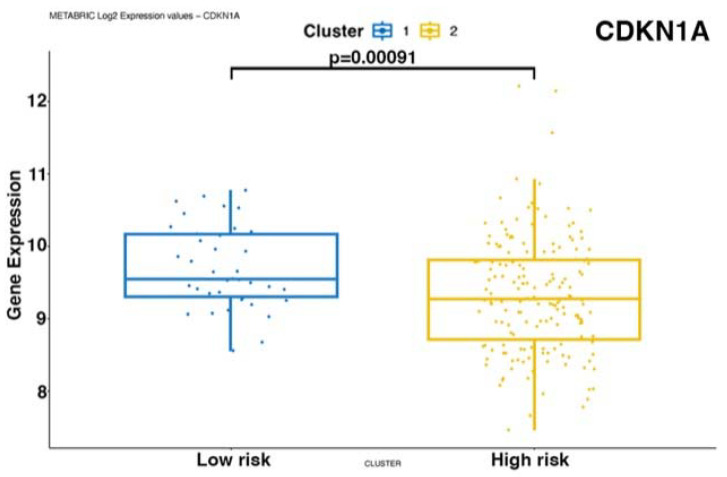
CDKN1A gene expression in the two risk-associated clusters in the METABRIC dataset. Cluster 1: high risk; Cluster 2: low risk.

To validate our findings on external cohorts, we analyzed the data from the TCGA [24] and METABRIC [25,26] studies. Unfortunately, these datasets do not provide data for mTNBC patients. Therefore, we used patient survival as a proxy for metastatic potential and prognosis. To this end, we considered TNBC patients, and we correlated their OS data with the RNA expression of *NOTCH-1* and genes associated with the *NOTCH-1* signaling such as *PIK3CA*, *AKT*, *c-MYC*, and *CDKN1A*. Consistent with the findings described in this study, *NOTCH-1* was identified as the main gene whose expression is associated with patient prognosis upon analysis of the TCGA (coefficient 0.077) (Figure 5). In the METABRIC dataset, *MYC*, *NOTCH-1*, and *PIK3CA* were significantly associated with prognosis, with regression coefficients of 0.149, 0.064, and 0.113, respectively (Figure 6). We then stratified the patients into two groups based on the expression levels of the genes significantly associated with prognosis. We evaluated the expression levels of the genes of interest in these clusters. In addition to confirming the predicted differences in *MYC*, *NOTCH-1*, and *PIK3CA* expression, we also identified a significant association between the expression of *AKT2* (Figure 7) and *CDKN1A* (Figure 8) and prognosis in the METABRIC cohort.

In conclusion, TCGA and METABRIC analysis indicated that reduced *NOTCH-1*, c-*MYC*, *PIK3CA*, and *AKT2* expression correlates with better survival in TNBC patients.

## 4. Discussion

TNBC represents a significant challenge in the realm of breast cancer treatment. Characterized by high recurrence rates and increased metastatic potential, [27] patients with TNBC face a significantly elevated risk of developing metastasis, with approximately 15% of them eventually developing brain metastases [28]. mTNBC further compounds the challenge, conferring a very poor prognosis with a median OS of 9 to 17 months, likely due to the very limited therapeutic options. Indeed, standard-of-care treatment relies on classic chemotherapy, since targeted therapies such as endocrine therapy or HER2-targeted therapies cannot be applied due to the lack of expression of their targets [28]. The availability of immune checkpoint inhibitors such as pembrolizumab in combination with chemotherapy for selected patients whose tumors express PD-L1 has resulted in substantial benefits in terms of progression-free survival and OS (4). Antibody–drug conjugates (ADCs) are constituted by antibodies that target tumor-specific antigens chemically linked to cytotoxic payloads that potently kill tumor cells and provide a new and important option for the treatment of TNBC. Recently, a Trop-2-directed ADC linked to topoisomerase I inhibitor (SN-38), named sacituzumab govitecan (IMMU-132, Trodelvy, SG,) has been approved for the treatment of TNBC showing a significant benefit in heavily pretreated mTNBC and is now available in this setting [6]. With the expansion of new targets and indications, a new era of targeted anticancer therapy in breast cancer has started [29].

Clinical trials conducted in patients with advanced breast cancer to evaluate the mCHT administration of one or more agents are encouraging, showing a response rate of around 30–44% and a clinical benefit rate of around 70%, and demonstrating that the mCHT represents an important therapeutic option for patients suffering from mTNBC [30,31]. In the VICTOR-6 multicenter retrospective cohort study, Cazzaniga et al. reported an ORR of 17.5% in patients with mTNBC receiving first-line mCHT and an ORR of 14.8% in those receiving second-line mCHT [28,32].

Although based on a small patient cohort, our study offers a valuable opportunity for directly comparing first-line and second-line mCHT. Our cohort study revealed an important difference in terms of OS depending on whether the patients were treated with mCHT in the first-line or the second-line. The median OS of patients treated with first-line mCHT is 27 months, while for patients treated with second-line mCHT it is 21 months. Furthermore, two of the 14 patients who received first-line mCHT are still alive. As reported by the Surveillance, Epidemiology, and End Results Program (SEER), the probability of surviving 5 years after diagnosis is 9.9% [95% CI: 7.7–12.4]. Commonly, patients with relapsed TNBC have an OS that, except for a few cases, does not exceed 12 months. Thus, despite the challenges, mCHT emerges as a promising treatment option for mTNBC.

In order to better understand the possible molecular targets of mCHT, we decided to characterize, at the molecular level, one patient from the first group who is still alive. In fact, given the heterogeneity of TNBC [33] and these various characteristics—including pharmacological properties, tumor cell characteristics, and environmental factors—influence the clinical responses, it is of paramount importance to unveil the biological characteristics of the patients who better respond to therapy and understand which therapeutic strategies may be more suitable for individual patients. In a scenario in which chemotherapy is supported by only a few other effective tools against this subtype of breast cancer, mCHT plays a role that is destined to be increasingly central in the treatment of patients affected by mTNBC, especially considering that tumors can develop resistance due to intrinsic and acquired factors or a combination of both [34].

Cancer stem cells (CSCs) represent another critical feature in the context of drug resistance and cancer metastasis, and clinical data reports that stemness biomarkers are associated with decreased OS [35]. Understanding the biology of CSC is crucial for developing therapies that can specifically target these cells. In the context of TNBC, the maintenance of stemness properties is often associated with aggressive tumor behavior, resistance to chemotherapy, and increased likelihood of metastasis. The deregulation of various stemness-related signaling pathways has been confirmed in patients with TNBC, indicating their targeting has recently come under development as a novel treatment option [36]. Among these, it has recently emerged that therapies targeting aberrant Notch signaling could represent a possible treatment approach for TNBC patients. For example, γ-secretase inhibitors (GSIs) act by preventing the cleavage of the active form of all Notch receptors, leading to apoptosis in TNBC cell lines [37]. Recently, it has also been demonstrated the involvement of NOTCH-1 in the invasion and migration phases that characterize the epithelial–mesenchymal transition (EMT) process in TNBC [38]. The authors of this study showed that *NOTCH-1* is negatively regulated by miR-3178, which is remarkably lower in TNBC than other subtypes of breast cancer. The lower levels of miR-3178 lead to raised NOTCH-1 activity followed by improved expression of Snail1, which eventually contributes to the regulation of EMT [39]. In line with these data, NOTCH-1 expression is higher in cisplatin-resistant MDA-MB-231 TNBC cells compared to parental cells, and this contributed to inducing chemoresistance through activation of the AKT pathway and promotion of EMT [40].

In accord with the implication of NOTCH-1 as a hallmark of TNBC [23], we investigated NOTCH-1 expression along with other associated molecular markers—such as AKT, c-MYC, OCT3/4, SOX2, and NANOG, p21, ki-67—in the disease progression of an exceptional long survivor mTNBC patient treated with mCHT in the first line. Comparing the biopsies taken at the diagnosis of mTNBC in 2019 and at relapse in 2023, we found that, among the stemness markers, only NOTCH-1 was remarkably modulated, whereas OCT3/4 and SOX2 tested negative in both biopsies. As reported in Figure 4A, NOTCH-1 immunoreactivity shifts from prevalent to decreased membrane expression in the two different moments; interestingly, the decreased membranous expression was accompanied by an increased cytoplasmic staining at relapse. As reported previously [41], the intensity and the localization of Notch signaling are crucial for cell fate decisions. In fact, Notch is constitutively internalized and recycled, which maintains the cells’ ability to respond to the ligand. Therefore, the reduction of the membrane recycling pathway is a fundamental step in the control of Notch signaling. Moreover, the described correlation between lower cytoplasmic staining of NOTCH-1 protein and adverse outcomes, such as cancer recurrence, bone metastasis, and worse disease-free survival, particularly in patients with estrogen receptor-positive ER/HER2+ cancers, has been reported, thus adding valuable context to the potential significance of NOTCH-1 in breast cancer [42]. It is important to note that while much of the research on NOTCH-1 in breast cancer has focused on its role in the context of the cell membrane and its involvement in signaling pathways, there is limited information specifically addressing its cytoplasmic role in breast cancer. What is well known is that membranous NOTCH can be cleaved, releasing the NICD that can be translocated into the nucleus (where it acts as a transcription factor) or remain in the cytoplasm to crosstalk with other signaling pathways, such as c-MYC, AKT, and others. Interestingly, our data showed that at a complete disappearance of the pAKT signal, as well as a significant decrease in c-MYC oncoprotein staining at relapse compared to what was observed at the diagnosis of mTNBC (Figure 4B,C, respectively), suggesting that the therapies administered to the patient had a significant impact in modulating cell survival and proliferation via negative regulation of pAKT, and cell cycle progression via the decrease of c-MYC, an oncoprotein often deregulated in cancer. Loss of pAKT signal and decreased c-MYC staining suggest that negative regulation of these pathways may contribute to cell growth inhibition through upregulation of the cell cycle inhibitor p21, which, in fact, increased in the post-treatments’ biopsy (Figure 4E). Accordingly, in the same biopsy, a remarkable decrease of the cell proliferation marker Ki-67 was observed (Figure 4D).

Notably, the importance of *NOTCH-1* and of genes associated with its pathway has been further supported by analyzing their expression in two large-scale external TNBC datasets (Figure 5, Figure 6, Figure 7 and Figure 8). In fact, our analysis confirmed that a lower expression of *NOTCH-1*, *MYC*, *AKT2*, and *PIK3CA*, together with increased *CDKN1A* expression, correlate with better OS.

**Figure 9:**
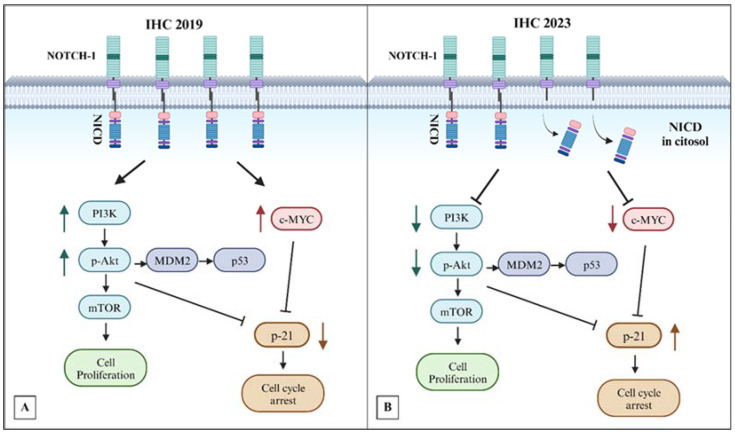
Proposed model of the role of NOTCH-1 and its molecular targets in response to mCHT therapy. (**A**) Increased NOTCH-1 at the membrane level leads to increased expression of c-MYC and pAKT and a decreased p21 expression, thus sustaining cell cycle entry and proliferation. (**B**) After therapy, the release of NOTCH-1 cytosolic domain into the intracellular environment reduces c-MYC and pAKT, with a consequent increase in p21 expression leading to cell cycle arrest. Created with BioRender.com. Agreement numbers YM26CPHPZZ for IHC 2019 (URL: https://app.biorender.com/illustrations/656535f574505e8f4467eb49, 8 January 2024) and SU26CPGIJX for IHC 2023 (URL: https://app.biorender.com/illustrations/6571aea4a99ab03d8c48772f, 8 January 2024).

Overall, our data led us to propose the schematic representation of the suggested relationship between NOTCH-1 signaling and its molecular targets before and after therapies, as depicted in Figure 9.

These results suggest that a complex interplay of signaling pathways is modulated in an exceptionally long survivor mTNBC patient treated with mCHT as first-line therapy followed by MTD. The disappearance of pAKT, the decrease in c-MYC, together with the upregulation of p21 and the decrease in Ki-67, collectively indicate that a potential shift toward reduced proliferation and cell cycle arrest occurred in the tumor following the indicated therapeutic combination.

## 5. Conclusions

These findings contribute insights into the molecular mechanisms of mCHT in a patient with mTNBC, offering potential biomarkers for personalized therapy. Expanding the molecular study to more patients would be advantageous to confirm our data and to find a molecular explanation of the efficacy of metronomic treatments.

## Figures and Tables

**Table 1:** Patients and disease characteristics at diagnosis of metastatic breast cancer treated with first- or second-line mCHT.

	First Line mCHT Treatment (N 14) N (%)	Second Line mCHT Treatment (N 13) N (%)
**Median age**—**yr [range]**	73 [42–79]	62 [41–74]
**Cardiovascular comorbidity**	2 (14.3)	1 (7.7)
**TNBC at first diagnosis**	11 (78.6)	8 (61.5)
**Previous neoadjuvant chemotherapy**	13 (92.8)	9 (69.2)
**No. of metastatic sites**		
1	11 (78.6)	13 (100)
≥2	3 (21.4)	-
**Sites of metastases**		
Brain	1 (7.1)	-
Bone	4 (28.6)	2 (15.4)
Visceral	7 (50)	6 (46.1)
Other	5 (35.7)	5 (38.5)

**Table 2:** The antibodies used to carry out immunohistochemical analysis.

Antibody	Source	Clonality	Company	Cat. Number
NOTCH-1	rabbit	monoclonal	Cell Signaling Technology	D6F11
pAKT	rabbit	monoclonal	Cell Signaling Technology	D25E6
c-MYC	rabbit	monoclonal	Cell Signaling Technology	D84C12
p21	rabbit	monoclonal	Cell Signaling Technology	12D1
SOX2	mouse	monoclonal	Santa Cruz Biotechnology	Sc-365823
OCT3/4	mouse	monoclonal	Santa Cruz Biotechnology	Sc-5279
NANOG	rabbit	monoclonal	Abcam	Ab214549
ER	rabbit	monoclonal	Dako	M3643
PR	mouse	monoclonal	Dako	M3568
HER2	rabbit	polyclonal	Dako	A0485
GATA3	mouse	monoclonal	Sigma-Aldrich	L50-823
Ki-67	mouse	monoclonal	Dako	GA626

## Data Availability

Data from TCGA and METABRIC studies were downloaded from cBioPortal respectively at https://www.cbioportal.org/study/summary?id=brca_tcga_pan_can_atlas_2018 and https://www.cbioportal.org/study/summary?id=brca_metabric.
